# Complex Problem Solving in Teams: The Impact of Collective Orientation on Team Process Demands

**DOI:** 10.3389/fpsyg.2017.01730

**Published:** 2017-09-29

**Authors:** Vera Hagemann, Annette Kluge

**Affiliations:** Business Psychology, Faculty of Psychology, Ruhr-University Bochum, Bochum, Germany

**Keywords:** interdependence, team processes, complex problem solving, collective orientation, trust, cohesion, C3Fire, microworld

## Abstract

Complex problem solving is challenging and a high-level cognitive process for individuals. When analyzing complex problem solving in teams, an additional, new dimension has to be considered, as teamwork processes increase the requirements already put on individual team members. After introducing an idealized teamwork process model, that complex problem solving teams pass through, and integrating the relevant teamwork skills for interdependently working teams into the model and combining it with the four kinds of team processes (transition, action, interpersonal, and learning processes), the paper demonstrates the importance of fulfilling team process demands for successful complex problem solving within teams. Therefore, results from a controlled team study within complex situations are presented. The study focused on factors that influence action processes, like coordination, such as emergent states like collective orientation, cohesion, and trust and that dynamically enable effective teamwork in complex situations. Before conducting the experiments, participants were divided by median split into two-person teams with either high (*n* = 58) or low (*n* = 58) collective orientation values. The study was conducted with the microworld C3Fire, simulating dynamic decision making, and acting in complex situations within a teamwork context. The microworld includes interdependent tasks such as extinguishing forest fires or protecting houses. Two firefighting scenarios had been developed, which takes a maximum of 15 min each. All teams worked on these two scenarios. Coordination within the team and the resulting team performance were calculated based on a log-file analysis. The results show that no relationships between trust and action processes and team performance exist. Likewise, no relationships were found for cohesion. Only collective orientation of team members positively influences team performance in complex environments mediated by action processes such as coordination within the team. The results are discussed in relation to previous empirical findings and to learning processes within the team with a focus on feedback strategies.

## Introduction

Complex problems in organizational contexts are seldom solved by individuals. Generally, interdependently working teams of experts deal with complex problems (Fiore et al., 2010), which are characterized by element interactivity/ interconnectedness, dynamic developments, non-transparency and multiple, and/or conflicting goals (Dörner et al., 1983; Brehmer, 1992; Funke, 1995). Complex problem solving “takes place for reducing the barrier between a given start state and an intended goal state with the help of cognitive activities and behavior. Start state, intended goal state, and barriers prove complexity, change dynamically over time, and can be partially intransparent” (Funke, 2012, p. 682). Teams dealing with complex problems in interdependent work contexts, for example in disaster, crisis or accident management, are called High Responsibility Teams. They are named High Responsibility Teams (HRTs; Hagemann, 2011; Hagemann et al., 2011) due to their dynamic and often unpredictable working conditions and demanding work contexts, in which technical faults and slips have severe consequences for human beings and the environment if they are not identified and resolved within the team immediately (Kluge et al., 2009). HRTs bear responsibility regarding lives of third parties and their own lives based on their actions and consequences.

The context of interdependently working HRTs, dealing with complex problems, is described as follows (Zsambok, 1997): Members of interdependently working teams have to reach ill-defined or competing goals in common in poor structured, non-transparent and dynamically changing situations under the consideration of rules of engagement and based on several cycles of joint action. Some or all goals are critical in terms of time and the consequences of actions result in decision-based outcomes with high importance for the culture (e.g., human life). In HRT contexts, added to the features of the complexity of the problem, is the complexity of relationships, which is called social complexity (Dörner, 1989/2003) or crew coordination complexity (Kluge, 2014), which results from the interconnectedness between multiple agents through coordination requirements. The dynamic control aspect of the continuous process is coupled with the need to coordinate multiple highly interactive processes imposing high coordination demands (Roth and Woods, 1988; Waller et al., 2004; Hagemann et al., 2012).

Within this article, it is important to us to describe the theoretical background of complex problem solving in teams in depth and to combine different but compatible theoretical approaches, in order to demonstrate their theoretical and practical use in the context of the analysis of complex problem solving in teams. In Industrial and Organizational Psychology, a detailed description of tasks and work contexts that are in the focus of the analysis is essential. The individual or team task is the point of intersection between organization and individual as a “psychologically most relevant part” of the working conditions (Ulich, 1995). Thus, the tasks and the teamwork context of teams that deal with complex problems is of high relevance in the present paper. We will comprehensively describe the context of complex problem solving in teams by introducing a model of an idealized teamwork process that complex problem solving teams pass through and extensively integrate the relevant teamwork skills for these interdependently working teams into the idealized teamwork process model.

Furthermore, we will highlight the episodic aspect concerning complex problem solving in teams and combine the agreed on transition, action, interpersonal and learning processes of teamwork with the idealized teamwork process model. Because we are interested in investigating teamwork competencies and action processes of complex problem solving teams, we will analyze the indirect effect of collective orientation on team performance through the teams' coordination behavior. The focusing of the study will be owed to its validity. Even though that we know that more aspects of the theoretical framework might be of interest and could be analyzed, we will focus on a detail within the laboratory experiment for getting reliable and valid results.

### Goal, task, and outcome interdependence in teamwork

Concerning interdependence, teamwork research focuses on three designated features, which are in accordance with general process models of human action (Hertel et al., 2004). One type is *goal* interdependence, which refers to the degree to which teams have distinct goals as well as a linkage between individual members and team goals (Campion et al., 1993; Wageman, 1995). A second type is *task* interdependence, which refers to the interaction between team members. The team members depend on each other for work accomplishment, and the actions of one member have strong implications for the work process of all members (Shea and Guzzo, 1987; Campion et al., 1993; Hertel et al., 2004). The third type is *outcome* interdependence, which is defined as the extent to which one team member's outcomes depend on the performance of other members (Wageman, 1995). Accordingly, the rewards for each member are based on the total team performance (Hertel et al., 2004). This can occur, for instance, if a team receives a reward based on specific performance criteria. Although interdependence is often the reason why teams are formed in the first place, and it is stated as a defining attribute of teams (Salas et al., 2008), different levels of task interdependence exist (Van de Ven et al., 1976; Arthur et al., 2005).

The workflow pattern of teams can be

1. Independent or pooled (activities are performed separately),
2. Sequential (activities flow from one member to another in a unidirectional manner),
3. Reciprocal (activities flow between team members in a back and forth manner) or
4. Intensive (team members must simultaneously diagnose, problem-solve, and coordinate as a team to accomplish a task).

Teams that deal with complex problems work within intensive interdependence, which requires greater coordination patterns compared to lower levels of interdependence (Van de Ven et al., 1976; Wageman, 1995) and necessitates mutual adjustments as well as frequent interaction and information integration within the team (Gibson, 1999; Stajkovic et al., 2009).

Thus, in addition to the cognitive requirements related to information processing (e.g., encoding, storage and retrieval processes (Hinsz et al., 1997), simultaneously representing and anticipating the dynamic elements and predicting future states of the problem, balancing contradictory objectives and decide on the right timing for actions to execute) of individual team members, the interconnectedness between the experts in the team imposes high team process demands on the team members. These team process demands follow from the required interdependent actions of all team members for effectively using all resources, such as equipment, money, time, and expertise, to reach high team performance (Marks et al., 2001). Examples for team process demands are the communication for building a shared situation awareness, negotiating conflicting perspectives on how to proceed or coordinating and orchestrating actions of all team members.

### A comprehensive model of the idealized teamwork process

**Figure 1:**
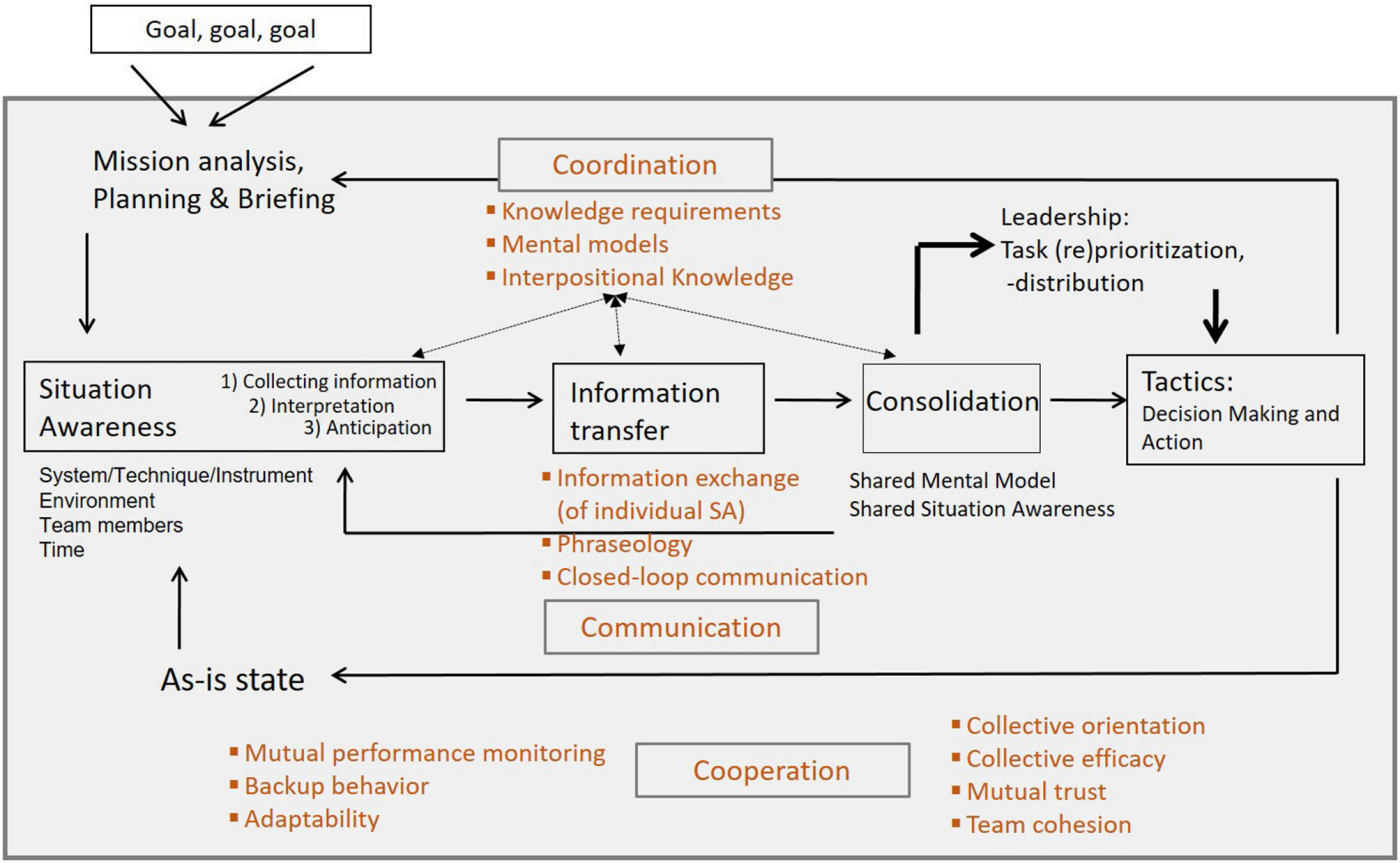
Relevant teamwork skills (orange color) for interdependently working teams (see Wilson et al., 2010) integrated into the model of an idealized teamwork process.

The cognitive requirements, that complex problem solving teams face, and the team process demands are consolidated within our model of an idealized teamwork process in Figure 1 (Hagemann, 2011; Kluge et al., 2014). Individual and team processes converge sequential and in parallel and influencing factors as well as process demands concerning complex problem solving in teams can be extracted. The core elements of the model are situation awareness, information transfer, individual and shared mental models, coordination and leadership, and decision making.

Complex problem solving teams are responsible for finding solutions and reaching specified goals. Based on the overall goals various sub goals will be identified at the beginning of the teamwork process in the course of mission analysis, strategy formulation and planning, all aspects of the *transition phase* (Marks et al., 2001). The transition phase processes occur during periods of time when teams focus predominantly on evaluation and/or planning activities. The identified and communicated goals within the team represent relevant input variables for each team member in order to build up a *Situation Awareness* (SA). SA contains three steps and is the foundation for an ideal and goal directed collaboration within a team (Endsley, 1999; Flin et al., 2008). The individual SA is the start and end within the idealized teamwork process model. SA means the assessment of a situation which is important for complex problem solving teams, as they work based on the division of labor as well as interdependently and each team member needs to achieve a correct SA and to share it within the team. Each single team member needs to utilize all technical and interpersonal resources in order to collect and interpret up-to-date goal directed information and to share this information with other team members via “closed-loop communication.”

This *information transfer* focuses on sending and receiving single SA between team members in order to build up a *Shared Situation Awareness* (SSA). Overlapping cuts of individual SA are synchronized within the team and a bigger picture of the situation is developed. Creating a SSA means sharing a common perspective of the members concerning current events within their environment, their meaning and their future development. This shared perspective enables problem-solving teams to attain high performance standards through corresponding and goal directed actions (Cannon-Bowers et al., 1993).

Expectations of each team member based on briefings, *individual mental models* and *interpositional knowledge* influence the SA, the information transfer and the consolidation process. Mental models are internal and cognitive representations of relations and processes (e.g., execution of tactics) between various aspects or elements of a situation. They help team members to describe, explain and predict circumstances (Mathieu et al., 2000). Mental models possess knowledge elements required by team members in order to assess a current situation in terms of SA. Interpositional knowledge refers to an individual understanding concerning the tasks and duties of all team members, in order to develop an understanding about the impact of own actions on the actions of other team members and vice versa. It supports the team in identifying the information needs and the amount of required help of other members and in avoiding team conflicts (Smith-Jentsch et al., 2001). This knowledge is the foundation for anticipating the team members' needs for information and it is important for matching information within the team.

Based on the information matching process within the team, a common understanding of the problem, the goals and the current situation is developed in terms of a *Shared Mental Model* (SMM), which is important for the subsequent decisions. SMM are commonly shared mental models within a team and refer to the organized knowledge structures of all team members, that are shared with each other and which enable the team to interact goal-oriented (Mathieu et al., 2000). SMM help complex problem solving teams during high workload to adapt fast and efficiently to changing situations (Waller et al., 2004). They also enhance the teams' performance and communication processes (Cannon-Bowers et al., 1993; Mathieu et al., 2000). Especially under time pressure and in crucial situations when overt verbal communication and explicit coordination is not applicable, SMM are fundamental in order to coordinate implicitly. This information matching process fosters the building of a shared understanding of the current situation and the required actions. In order to do so teamwork skills (see Wilson et al., 2010) such as *communication, coordination*, and *cooperation* within the team are vitally important. Figure 1 incorporates the teamwork skills into the model of an idealized teamwork process.

Depending on the shared knowledge and SA within the team, the coordination can be based either on well-known procedures or shared expectations within the team or on explicit communication based on task specific phraseology or closed-loop communication. Cooperation needs mutual performance monitoring within the team, for example, in order to apply task strategies to accurately monitor teammate performance and prevent errors (Salas et al., 2005). Cooperation also needs backup behavior of each team member, for example, and continuous actions in reference to the collective events. The anticipation of other team members' needs under high workload maintains the teams' performance and the well-being of each team member (Badke-Schaub, 2008). A successful pass through the teamwork process model also depends e.g., on the trust and the cohesion within the team and the collective orientation of each team member.

Collective orientation (CO) is defined “as the propensity to work in a collective manner in team settings” (Driskell et al., 2010, p. 317). Highly collectively oriented people work with others on a task-activity and team-activity track (Morgan et al., 1993) in a goal-oriented manner, seek others' input, contribute to team outcomes, enjoy team membership, and value cooperativeness more than power (Driskell et al., 2010). Thus, teams with collectively oriented members perform better than teams with non-collectively oriented members (Driskell and Salas, 1992). CO, trust and cohesion as well as other coordination and cooperation skills are so called emergent sates that represent cognitive, affective, and motivational states, and not traits, of teams and team members, and which are influenced, for example, by team experience, so that emergent states can be considered as team inputs but also as team outcomes (Marks et al., 2001).

Based on the information matching process the complex problem solving team or the team leader needs to make *decisions* in order to execute actions. The *task prioritization* and *distribution* is an integrated part of this step (Waller et al., 2004). Depending on the progress of the dynamic, non-transparent and heavily foreseeable situation tasks have to be re-prioritized during episodes of teamwork. Episodes are “temporal cycles of goal-directed activity” in which teams perform (Marks et al., 2001, p. 359). Thus, the team acts adaptive and is able to react flexible to situation changes. The team coordinates implicitly when each team member knows what he/she has to do in his/her job, what the others expect from him/her and how he/she interacts with the others. In contrast, when abnormal events occur and they are recognized during SA processes, the team starts coordinating explicitly via communication, for example. Via closed-loop communication and based on interpositional knowledge new strategies are communicated within the team and tasks are re-prioritized.

The result of the decision making and action taking flows back into the individual SA and the as-is state will be compared with the original goals. This model of an idealized teamwork process (Figure 1) is a regulator circuit with feedback loops, which enables a team to adapt flexible to changing environments and goals. The foundation of this model is the classic Input-Process-Outcome (IPO) framework (Hackman, 1987) with a strong focus on the process part. IPO models view processes as mechanisms linking variables such as member, team, or organizational features with outcomes such as performance quality and quantity or members' reactions. This mediating mechanism, the *team process*, can be defined as “members' interdependent acts that convert inputs to outcomes through cognitive, verbal, and behavioral activities directed toward organizing taskwork to achieve collective goals” (Marks et al., 2001, p. 357). That means team members interact interdependently with other members as well as with their environment. These cognitive, verbal, and behavioral activities directed toward taskwork and goal attainment are represented as gathering situation awareness, communication, coordination, cooperation, the consolidation of information, and task prioritization within our model of an idealized teamwork process. Within the context of complex problem solving, teams have to face team process demands in addition to cognitive challenges related to individual information processing. That means teamwork processes and taskwork to solve complex problems co-occur, the processes guide the execution of taskwork.

**Figure 2:**
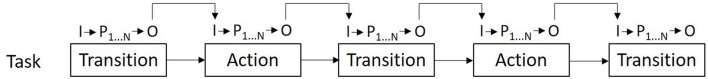
Teamwork episodes with repetitive IPO cycles (Marks et al., 2001).

The dynamic nature of teamwork and temporal influences on complex problem solving teams are considered within adapted versions (Marks et al., 2001; Ilgen et al., 2005) of the original IPO framework. These adaptations propose that teams experience cycles of joint action, so called episodes, in which teams perform and also receive feedback for further actions. The IPO cycles occur sequentially and simultaneously and are nested in transition and action phases within episodes in which outcomes from initial episodes serve as inputs for the next cycle (see Figure 2). These repetitive IPO cycles are a vital element of our idealized teamwork process model, as it incorporates feedback loops in such a way, that the outcomes, e.g., changes within the as-is state, are continuously compared with the original goals. Detected discrepancies within the step of updating SA motivate the team members to consider further actions for goal accomplishment.

When applying this episodic framework to complex problem solving teams it becomes obvious that teams handle different types of taskwork at different phases of task accomplishment (Marks et al., 2001). That means episodes consist of two phases, so-called *action* and *transition phases*, in which teams are engaged in activities related to goal attainment and in other time in reflecting on past performance and planning for further common actions. The addition of the social complexity to the complexity of the problem within collaborative complex problem solving comes to the fore here. During transition phases teams evaluate their performance, compare the as-is state against goals, reflect on their strategies and plan future activities to guide their goal accomplishment. For example, team members discuss alternative courses of action, if their activities for simulated firefighting, such as splitting team members in order to cover more space of the map, are not successful. During action phases, teams focus directly on the taskwork and are engaged in activities such as exchanging information about the development of the dynamic situation or supporting each other. For example, a team member recognizes high workload of another team member and supports him/her in collecting information or in taking over the required communication with other involved parties.

### Transition and action phases

The idealized teamwork process model covers these transition and action phases as well as the processes occurring during these two phases of team functioning, which can be clustered into transition, action, and interpersonal processes. That means during complex problem solving the relevant or activated teamwork processes in the transition and action phases change as teams move back and forth between these phases. As this taxonomy of team processes from Marks et al. (2001) states that a team process is multidimensional and teams use different processes simultaneously, some processes can occur either during transition periods or during action periods or during both periods. *Transition processes* especially occur during transition phases and enable the team to understand their tasks, guide their attention, specify goals and develop courses of action for task accomplishment. Thus, transition processes include (see Marks et al., 2001) mission analysis, formulation and planning (Prince and Salas, 1993), e.g., fighting a forest fire, goal specification (Prussia and Kinicki, 1996), e.g., saving as much houses and vegetation as possible, and strategy formulation (Prince and Salas, 1993; Cannon-Bowers et al., 1995), e.g., spreading team members into different geographic directions. *Action processes* predominantly occur during action phases and support the team in conducting activities directly related to goal accomplishment. Thus, action processes are monitoring progress toward goals (Cannon-Bowers et al., 1995), e.g., collecting information how many cells in a firefighting simulation are still burning, systems monitoring (Fleishman and Zaccaro, 1992), e.g., tracking team resources such as water for firefighting, team monitoring and backup behavior (Stevens and Campion, 1994; Salas et al., 2005), e.g., helping a team member and completing a task for him/her, and coordination (Fleishman and Zaccaro, 1992; Serfaty et al., 1998), e.g., orchestrating the interdependent actions of the team members such as exchanging information during firefighting about positions of team members for meeting at the right time at the right place in order to refill the firefighters water tanks. Especially the coordination process is influenced by the amount of task interdependence as coordination becomes more and more important for effective team functioning when interdependence increases (Marks et al., 2001). *Interpersonal processes* occur during transition and action phases equally and lay the foundation for the effectiveness of other processes and govern interpersonal activities (Marks et al., 2001). Thus, interpersonal processes include conflict management (Cannon-Bowers et al., 1995), like the development of team rules, motivation and confidence building (Fleishman and Zaccaro, 1992), like encourage team members to perform better, and affect management (Cannon-Bowers et al., 1995), e.g., regulating member emotions during complex problem solving.

**Figure 3:**
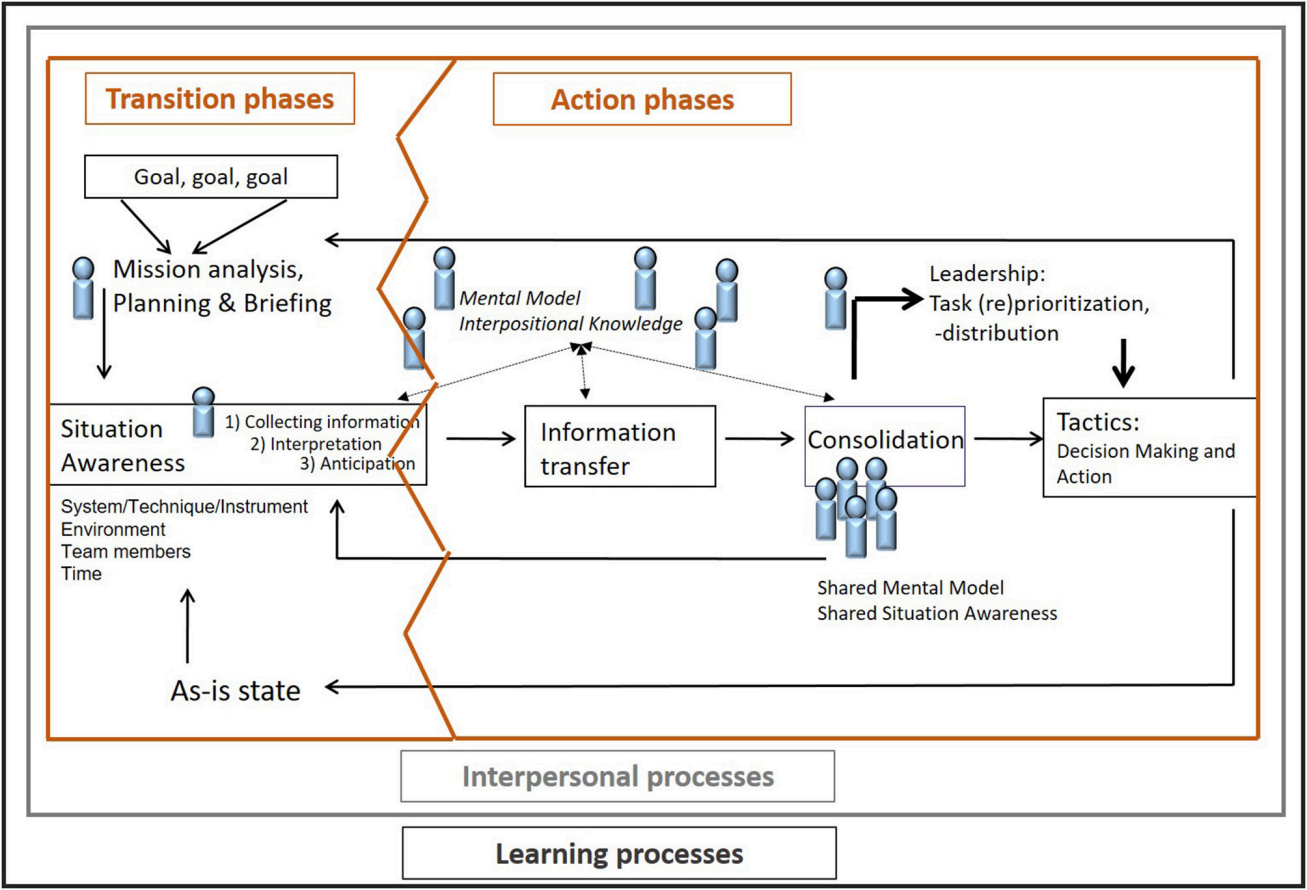
The integration of transition, action, interpersonal, and learning processes into the model of an idealized teamwork process.

Summing up, process demands such as transition processes that complex problem solving teams pass through, are mission analysis, planning, briefing and goal specification, visualized on the left side of the idealized teamwork process model (see Figure 3). The results of these IPO cycles lay the foundation for gathering a good SA and initiating activities directed toward taskwork and goal accomplishment and therefore initiating action processes. The effective execution of action processes depends on the communication, coordination, cooperation, matching of information, and task prioritization as well as emergent team cognition variables (SSA and SMM) within the team. The results, like decisions, of these IPO cycles flow back into the next episode and may initiate further transition processes. In addition, interpersonal processes play a crucial role for complex problem solving teams. That means, conflict management, motivating and confidence building, and affect management are permanently important, no matter whether a team runs through transition or action phases and these interpersonal processes frame the whole idealized teamwork process model. Therefore, interpersonal processes are also able to impede successful teamwork at any point as breakdowns in conflict or affect management can lead to coordination breakdowns (Wilson et al., 2010) or problems with monitoring or backing up teammates (Marks et al., 2001). Thus, complex problem solving teams have to face these multidimensional team process demands in addition to cognitive challenges, e.g., information storage or retrieval (Hinsz et al., 1997), related to individual information processing.

### Team learning opportunities for handling complex problems

In order to support teams in handling complex situations or problems, learning opportunities seem to be very important for successful task accomplishment and for reducing possible negative effects of team process demands. Learning means any kind of relative outlasted changes in potential of human behavior that cannot be traced back to age-related changes (Bower and Hilgard, 1981; Bredenkamp, 1998). Therefore, Schmutz et al. (2016) amended the taxonomy of team processes developed by Marks et al. (2001) and added learning processes as a fourth category of processes, which occur during transition and action phases and contribute to overall team effectiveness. *Learning processes* (see also Edmondson, 1999) include observation, e.g., observing own and other team members' actions such as the teammate's positioning of firewalls in order to protect houses in case of firefighting, feedback, like giving a teammate information about the wind direction for effective positioning of firewalls, and reflection, e.g., talking about procedures for firefighting or refilling water tanks, for example, within the team. Learning from success and failure and identifying future problems is crucial for the effectiveness of complex problem solving teams and therefore possibilities for learning based on repetitive cycles of joint action or episodes and reflection of team members' activities during action and transition phases should be used effectively (Edmondson, 1999; Marks et al., 2001). The processes of the idealized teamwork model are embedded into these learning processes (see Figure 3).

The fulfillment of transition, action, interpersonal and learning processes contribute significantly to successful team performance in complex problem solving. For clustering these processes, transition and action processes could be seen as operational processes and interpersonal and learning process as support processes. When dealing with complex and dynamic situations teams have to face these team process demands more strongly than in non-complex situations. For example, goal specification and prioritization or strategy formulation, both aspects of transition processes, are strongly influenced by multiple goals, interconnectedness or dynamically and constantly changing conditions. The same is true for action processes, such as monitoring progress toward goals, team monitoring and backup behavior or coordination of interdependent actions. Interpersonal processes, such as conflict and affect management or confidence building enhance the demands put on team members compared to individuals working on complex problems. Interpersonal processes are essential for effective teamwork and need to be cultivated during episodes of team working, because breakdowns in confidence building or affect management can lead to coordination breakdowns or problems with monitoring or backing up teammates (Marks et al., 2001). Especially within complex situations aspects such as interdependence, delayed feedback, multiple goals and dynamic changes put high demands on interpersonal processes within teams. Learning processes, supporting interpersonal processes and the result of effective teamwork are e.g., observation of others' as well as own actions and receiving feedback by others or the system and are strongly influenced by situational characteristics such as non-transparency or delayed feedback concerning actions. It is assumed that amongst others team learning happens through repetitive cycles of joint action within the action phases and reflection of team members within the transition phases (Edmondson, 1999; Gabelica et al., 2014; Schmutz et al., 2016). The repetitive cycles help to generate SMM (Cannon-Bowers et al., 1993; Mathieu et al., 2000), SSA (Endsley and Robertson, 2000) or transactive memory systems (Hollingshead et al., 2012) within the team.

### Emergent states in complex team work and the role of collective orientation

IPO models propose that *input variables* and *emergent states* are able to influence team processes and therefore outcomes such as team performance positively. Emergent states represent team members' attitudes or motivations and are “properties of the team that are typically dynamic in nature and vary as a function of team context, inputs, processes, and outcomes” (Marks et al., 2001, p. 357). Both emergent states and interaction processes are relevant for team effectiveness (Kozlowski and Ilgen, 2006).

Emergent states refer to conditions that underlie and dynamically enable effective teamwork (DeChurch and Mesmer-Magnus, 2010) and can be differentiated from team process, which refers to interdependent actions of team members that transform inputs into outcomes based on activities directed toward task accomplishment (Marks et al., 2001). Emergent states mainly support the execution of behavioral processes (e.g., planning, coordination, backup behavior) during the action phase, meaning during episodes when members are engaged in acts that focus on task work and goal accomplishment. Emergent states like trust, cohesion and CO are “products of team experiences (including team processes) and become new inputs to subsequent processes and outcomes” (Marks et al., 2001, p. 358). Trust between team members and cohesion within the team are emergent states that develop over time and only while experiencing teamwork in a specific team. CO is an emergent state that a team member brings along with him/her into the teamwork, is assumed to be more persistent than trust and cohesion, and can, but does not have to, be positively and negatively influenced by experiencing teamwork in a specific team for a while or by means of training (Eby and Dobbins, 1997; Driskell et al., 2010). Thus, viewing emergent states on a continuum, trust and cohesion are assumed more fluctuating than CO, but CO is much more sensitive to change and direct experience than a stable trait such as a personality trait.

CO of team members is one of the teamwork-relevant competencies that facilitates team processes, such as collecting and sharing information between team members, and positively affects the success of teams, as people who are high in CO work with others in a goal-oriented manner, seek others' input and contribute to team outcomes (Driskell et al., 2010). CO is an emergent state, as it can be an input variable as well as a teamwork outcome. CO is context-dependent, becomes visible in reactions to situations and people, and can be influenced by experience (e.g., individual learning experiences with various types of teamwork) or knowledge or training (Eby and Dobbins, 1997; Bell, 2007). CO enhances team performance through activating transition and action processes such as coordination, evaluation and consideration of task inputs from other team members while performing a team task (Driskell and Salas, 1992; Salas et al., 2005). Collectively oriented people effectively use available resources in due consideration of the team's goals, participate actively and adapt teamwork processes adequately to the situation.

Driskell et al. (2010) and Hagemann (2017) provide a sound overview of the evidence of discriminant and convergent validity of CO compared to other teamwork-relevant constructs, such as cohesion, also an emergent state, or cooperative interdependence or preference for solitude. Studies analyzing collectively and non-collectively oriented persons' decision-making in an interdependent task demonstrated that teams with non-collectively oriented members performed poorly in problem solving and that members with CO judged inputs from teammates as more valuable and considered these inputs more frequently (Driskell and Salas, 1992). Eby and Dobbins (1997) also showed that CO results in increased coordination among team members, which may enhance team performance through information sharing, goal setting and strategizing (Salas et al., 2005). Driskell et al. (2010) and Hagemann (2017) analyzed CO in relation to team performance and showed that the effect of CO on team performance depends on the task type (see McGrath, 1984). Significant positive relationships between team members' CO and performance were found in relation to the task types choosing/decision making and negotiating (Driskell et al., 2010) respectively choosing/decision making (Hagemann, 2017). These kinds of tasks are characterized by much more interdependence than task types such as executing or generating tasks. As research shows that the positive influence of CO on team performance unfolds especially in interdependent teamwork contexts (Driskell et al., 2010), which require more team processes such as coordination patterns (Van de Ven et al., 1976; Wageman, 1995) and necessitate mutual adjustments as well as frequent information integration within the team (Gibson, 1999; Stajkovic et al., 2009), CO might be vitally important for complex problem solving teams. Thus, CO as an emergent state of single team members might be a valuable resource for enhancing the team's performance when exposed to solving complex problems. Therefore, it will be of interest to analyze the influence of CO on team process demands such as coordination processes and performance within complex problem solving teams. We predict that the positive effect of CO on team performance is an indirect effect through coordination processes within the team, which are vitally important for teams working in intensive interdependent work contexts.

Hypothesis 1: CO leads to a better coordination behavior, which in turn leads to a higher team performance.

As has been shown in team research that emergent states like trust and cohesion (see also Figure 1) affect team performance, these two constructs are analyzed in conjunction with CO concerning action processes, such as coordination behavior and team performance. Trust between team members supports information sharing and the willingness to accept feedback, and therefore positively influences teamwork processes (McAllister, 1995; Salas et al., 2005). Cohesion within a team facilitates motivational factors and group processes like coordination and enhances team performance (Beal et al., 2003; Kozlowski and Ilgen, 2006).

Hypothesis 2: Trust shows a positive relationship with (a) action processes (team coordination) and with (b) team performance.
Hypothesis 3: Cohesion shows a positive relationship with (a) action processes (team coordination) and with (b) team performance.

## Materials and methods

In order to demonstrate the importance of team process demands for complex problem solving in teams, we used a computer-based microworld in a laboratory study. We analyzed the effectiveness of complex problem solving teams while considering the influence of input variables, like collective orientation of team members and trust and cohesion within the team, on action processes within teams, like coordination.

### The microworld for investigating teams process demands

**Figure 4:**
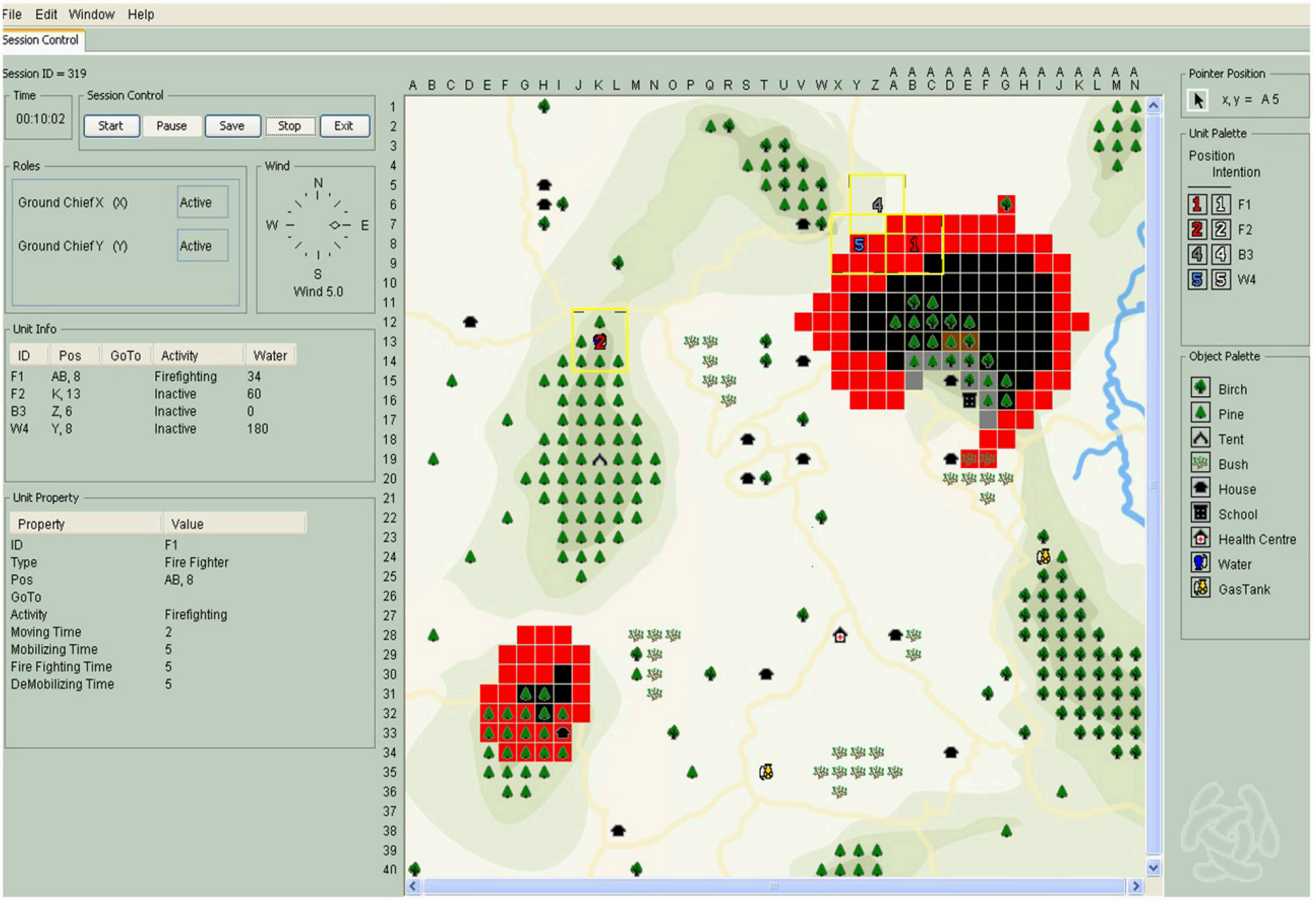
Examples for the complexity characteristics in Table 1 represented within a simulation scenario in C^3^Fire.

We used the simulation-based team task C^3^Fire (Granlund et al., 2001; Granlund and Johansson, 2004), which is described as an intensive interdependence team task for complex problem solving (Arthur et al., 2005). C^3^Fire is a command, control and communications simulation environment that allows teams' coordination and communication in complex and dynamic environments to be analyzed. C^3^Fire is a microworld, as important characteristics of the real world are transferred to a small and well-controlled simulation system. The task environment in C^3^Fire is complex, dynamic and opaque (see Table 1) and therefore similar to the cognitive tasks people usually encounter in real-life settings, in and outside their work place (Brehmer and Dörner, 1993; Funke, 2001). Figure 4 demonstrates how the complexity characteristics mentioned in Table 1 are realized in C^3^Fire. The screenshot represents the simulation manager's point of view, who is able to observe all units and actions and the scenario development. For more information about the units and scenarios, please (see the text below and the Supplementary Material). Complexity requires people to consider a number of facts. Because executed actions in C^3^Fire influence the ongoing process, the sequencing of actions is free and not stringent, such as a fixed (if X then Y) or parallel (if X then Y and Z) sequence (Ormerod et al., 1998). This can lead to stressful situations. Taking these characteristics of microworlds into consideration, team processes during complex problem solving can be analyzed within laboratories under controlled conditions. Simulated microworlds such as C^3^Fire allow the gap to be bridged between laboratory studies, which might show deficiencies regarding ecological validity, and field studies, which have been criticized due to their small amount of control (see Brehmer and Dörner, 1993).

**Table 1:** Overview of complexity characteristics of microworlds in general and in C^3^Fire (cf. Funke, 2001).

**Complexity**	**General**	**C^3^Fire examples**	**Representation in Figure 4**
Goals	People try to reach many goals, some of which may be contradictory, and therefore they have to make trade-offs.	Extinguish a forest fire and/or protect houses simultaneously.	Two fires are spreading out. Brown cells are extinguished, black cells are burned down. A house and a school are blocked with fire-breaks (gray cells).
Side-effects	Side effects of a given course of action exist due to coupled processes and force people to choose between many possible courses of action.	If the participant decides to refill his/her water tank on his/her back, he/she is not able to fight a fire during this refill process.	Unit 2, one firefighting unit, stands on the local water tank for refilling its water supply.
Dynamic	Microworlds are dynamic, because “their current state is a function of the history of the interaction between the subject and the system” and “they change, both as a consequence of the subject's actions and autonomously” (Brehmer and Dörner, 1993, p. 173). People have to act in real time and directly influence the system's state even though they do not know exactly when they have to make decisions.	If the participant does nothing, the fire spreads in all directions. If the participant extinguishes burning fields, the fire spreads in the directions where no firefighting occurs. If the wind direction changes, the direction of fire spreading also changes and the participant needs to recognize this for his/her further actions.	Two fires are spreading out into all directions. The fire stops bevor a placed fire-break. The fire spreads out predominantly in a westward direction, because the wind is coming from the East.
Opaque	Opaque means that the people do not have all relevant information. Thus, people have to form hypotheses and test them autonomously during activity.	Restricted visibility field. Not everything within the simulation environment is visible for the participants without exploring the environment. All units see the houses, trees, bushes and so on, but they can only see the fire if they are close to it.	The restricted visibility field is represented by the yellow squares. e.g., unit 5 only sees five burning cells and four non-burning cells and has an intersection of two cells with unit 4. Unit 1 only sees eight burning cells and one burned-out cell and has an intersection of one cell with unit 4.

In C^3^Fire, the teams' task is to coordinate their actions to extinguish a forest fire whilst protecting houses and saving lives. The team members' actions are interdependent. The simulation includes, e.g., forest fires, houses, tents, gas tanks, different kinds of vegetation and computer-simulated agents such as firefighting units (Granlund, 2003). It is possible, for example, that the direction of wind will change during firefighting and the time until different kinds of vegetation are burned down varies between those. In the present study, two simulation scenarios were developed for two-person teams and consisted of two firefighting units, one mobile water tank unit (responsible for re-filling the firefighting units' water tanks that contain a predefined amount of water) and one fire-break unit (a field defended with a fire-break cannot be ignited; the fire spreads around its ends). The two developed scenarios lasted for 15 min maximum. Each team member was responsible for two units in each scenario; person one for firefighting and water tank unit and person two for firefighting and fire-break unit. The user interface was a map system (40 × 40 square grid) with all relevant geographic information and positions of all symbols representing houses, water tank units and so on. All parts of the map with houses and vegetation were visible for the subjects, but not the fire itself or the other units; instead, the subjects were close to them with their own units (restricted visibility field; 3 × 3 square grid). The simulation was run on computers networked in a client-server configuration. The subjects used a chat system for communication that was logged. For each scenario, C^3^Fire creates a detailed log file containing all events that occurred over the course of the simulation. Examples of the C^3^Fire scenarios are provided in the Figures S1–3 and a short introduction into the microworld is given in the video. Detailed information regarding the scenario characteristics are given in Table S1. From scenario one to two, the complexity and interdependence increased.

### Participants

The study was conducted from Mai 2014 until March 2015. Undergraduate and graduate students (*N* = 116) studying applied cognitive sciences participated in the study (68.1% female). Their mean age was 21.17 years (*SD* = 3.11). Participants were assigned to 58 two-person teams, with team assignments being based on the pre-measured CO values (see procedure). They received 2 hourly credits as a trial subject and giveaways such as pencils and non-alcoholic canned drinks. The study was approved by the university's ethics committee in February 2014.

### Procedure

**Figure 5:**
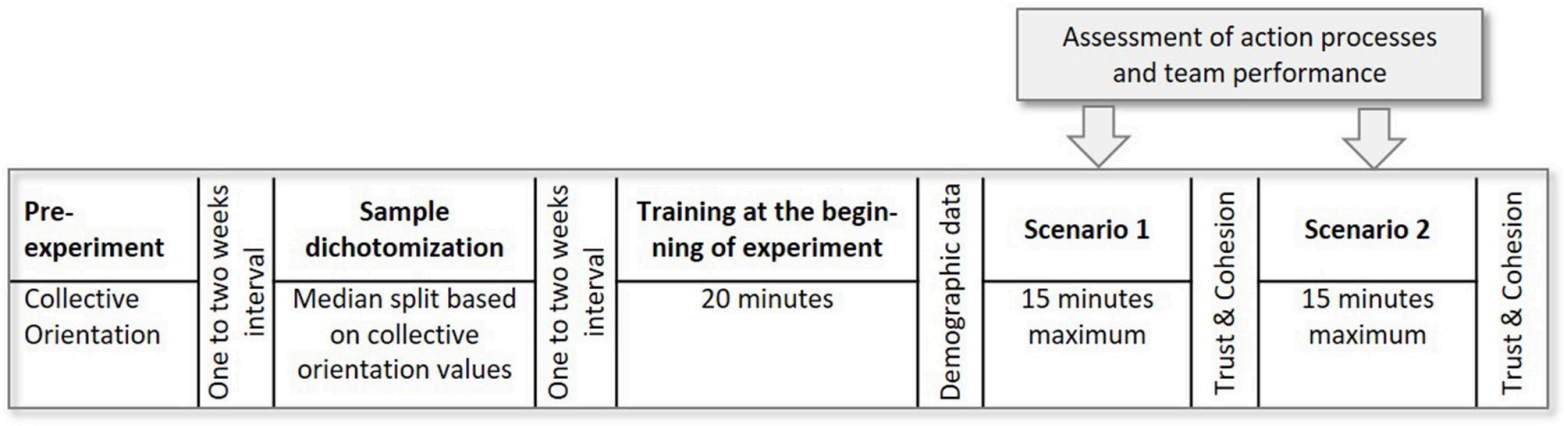
Overview about the procedure and measures.

The study was conducted within a laboratory setting at a university department for business psychology. Prior to the experiment, the participants filled in the CO instrument online and gave written informed consent (see Figure 5). The median was calculated subsequently (*Md* = 3.12; range: 1.69–4.06; scale range: 1–5) relating to the variable CO and two individuals with either high (*n* = 58) or low (*n* = 58) CO values were randomly matched as teammates. The matching process was random in part, as those two subjects were matched to form a team, whose preferred indicated time for participation in a specific week during data collection were identical. The participants were invited to the experimental study by e-mail 1–2 weeks after filling in the CO instrument. The study began with an introduction to the experimental procedure and the teams' task. The individuals received time to familiarize themselves with the simulation, received 20 min of training and completed two practice trials. After the training, participants answered a questionnaire collecting demographic data. Following this, a simulation scenario started and the participants had a maximum of 15 min to coordinate their actions to extinguish a forest fire whilst protecting houses and saving lives. After that, at measuring time T1, participants answered questionnaires assessing trust and cohesion within the team. Again, the teams worked on the following scenario 2 followed by a last round of questionnaires assessing trust and cohesion at T2.

### Measures

**Demographic data** such as age, sex, and study course were assessed after the training at the beginning of the experiment.

**Collective Orientation** was measured at an individual level with 16 items rated on a 5-point Likert scale (1 = *strongly disagree* to 5 = *strongly agree*) developed by the authors (Hagemann, 2017) based on the work of Driskell et al. (2010). The factorial structure concerning the German-language CO scale was proven prior to this study (χ^2^ = 162.25, *df* = 92, *p* = 0.000, χ^2^/df = 1.76, CFI = 0.97, TLI = 0.96, RMSEA = 0.040, CI = 0.030-0.051, SRMR = 0.043) and correlations for testing convergent and discriminant evidence of validity were satisfying. For example, CO correlated *r* = 0.09 (*p* > 0.10) with cohesion, *r* = 0.34 (*p* < 0.01) with cooperative interdependence and *r* = −0.28 (*p* < 0.01) with preference for solitude (Hagemann, 2017). An example item is “*I find working on team projects to be very satisfying*”. Coefficient alpha for this scale was 0.81.

**Trust** in team members' integrity, trust in members' task abilities and trust in members' work-related attitudes (Geister et al., 2006) was measured with seven items rated on a 5-point Likert scale (1 = *strongly disagree* to 5 = *strongly agree*). An example item is “*I can trust that I will have no additional demands due to lack of motivation of my team member*.” Coefficient alpha for this scale was 0.83 (T1) and 0.87 (T2).

**Cohesion** was measured with a six-item scale from Riordan and Weatherly (1999) rated on a 5-point Likert scale (1 = *strongly disagree* to 5 = *strongly agree*). An example item is “*In this team, there is a lot of team spirit among the members*.” Coefficient alpha for this scale was 0.87 (T1) and 0.87 (T2).

#### Action process: coordination

Successful coordination requires mechanisms that serve to manage dependencies between the teams' activities and their resources. Coordination effectiveness was assessed based on the time the firefighting units spent without water in the field in relation to the total scenario time. This measure is an indicator of the effectiveness of resource-oriented coordination, as it reflects an efficient performance regarding the water refill process in C^3^Fire, which requires coordinated actions between the two firefighting units and one water tank unit (Lafond et al., 2011). The underlying assumption is that a more successful coordination process leads to fewer delays in conducting the refill process. Coordination was calculated by a formula and values ranged between 0 and 1, with lower values indicating better coordination in the team (see Jobidon et al., 2012).

$$\begin{array}{l} Coordination=\text{time spent without water}\\ /\text{total time spent in scenario} \end{array}$$

#### Team performance

This measure related to the teams' goals (limiting the number of burned out cells and saving as many houses/buildings as possible) and was quantified as the number of protected houses and the number of protected fields and bushes/trees in relation to the number of houses, fields, and bushes/trees, respectively, which would burn in a worst case scenario. This formula takes into account that teams needing more time for firefighting also have more burning cells and show a less successful performance than teams that are quick in firefighting. To determine the worst case scenario, both 15-min scenarios were run with no firefighting action taken. Thus, the particularities (e.g., how many houses would burn down if no action was taken) of each scenario were considered. Furthermore, the houses, bushes/trees and fields were weighted according to their differing importance, mirroring the teams' goals. Houses should be protected and were most important. Bushes/trees (middle importance) burn faster than fields (lowest importance) and foster the expansion of the fire. Values regarding team performance ranged between 0 and 7.99, with higher values indicating a better overall performance. Team performance was calculated as follows (see Table 2):

$$\begin{array}{l} TeamPerformance=\left(\left(a/\max a\right)*5\right)+\left(\left(b/\max b\right)*2\right)\\ \text{ +}\left(\left(c/\text{max}c\right)*1\right) \end{array}$$

**Table 2:** Explanation of formula for calculating team performance in both scenarios.

**Symbol**		**Explanation**
a	=	number of protected houses (those that were not touched by fire)
b	=	number of protected bushes/trees
c	=	number of protected fields
max a	=	number of affected houses in the worst case (those that are burned out, extinguished or still on fire)
max b	=	number of affected bushes/trees in the worst case
max c	=	number of affected fields in the worst case
5	=	weighting of houses (highest priority)
2	=	weighting of bushes/trees (middle priority)
1	=	weighting of fields (lowest priority)

## Results

Means, standard deviations, internal consistencies, and correlations for all study variables are provided in Table 3.

**Table 3:** Means, standard deviations, internal consistencies, and correlations for all study variables.

	***M***	***SD***	**α**	**1**	**2**	**3**	**4**	**5**	**6**	**7**	**8**
1 Performance scenario 1	5.82	2.03		1							
2 Performance scenario 2	5.31	2.53		0.31^**^	1						
3 Time without water scenario 1	0.177	0.09		−0.48^**^	−0.24^**^	1					
4 Time without water scenario 2	0.214	0.10		−0.02	−0.30^**^	0.25^**^	1				
5 Collective Orientation	3.12	0.46	0.81	0.14	0.20^*^	−0.20^*^	−0.42^**^	1			
6 Trust T1	4.43	0.51	0.83	0.18	0.06	−0.11	−0.08	0.05	1		
7 Trust T2	4.47	0.50	0.87	−0.02	0.06	−0.00	−0.12	−0.03	0.83^**^	1	
8 Cohesion T1	4.02	0.64	0.87	0.00	−0.09	−0.22^*^	−0.06	−0.17	0.47^**^	0.51^**^	1
9 Cohesion T2	4.01	0.65	0.87	0.01	−0.07	−0.17	−0.08	−0.18	0.39^**^	0.47^**^	0.87^**^

*Performance range from 0 to 7.99; Time without Water range from 0 to 1 (lower values indicate a more effective handling of water); CO range from 1 to 5*.

* *p < 0.05*,

** *p < 0.01*.

Team complex problem solving in scenario 1 correlated significantly negative with time without water in scenario 1, indicating that a high team performance is attended by the coordination behavior (as a team process). The same was true for scenario 2. In addition, time without water as an indicator for team coordination correlated significantly negative with the team members' CO, indicating that team members with high CO values experience less time without water in the microworld than teams with members with low CO values.

In order to analyze the influence of CO on team process demands such as coordination processes and thereby performance within complex problem solving teams we tested whether CO would show an indirect effect on team performance through the teams' coordination processes. To analyze this assumption, indirect effects in simple mediation models were estimated for both scenarios (see Preacher and Hayes, 2004). The mean for CO was 3.44 (*SD* = 0.32) for teams with high CO values and it was 2.79 (*SD* = 0.35) for teams with low CO values. The mean concerning team performance in scenario 1 for teams with high CO values was 6.30 (*SD* = 1.64) and with low CO values 5.35 (*SD* = 2.30). The mean concerning time without water (coordination behavior) for teams with high CO values was 0.16 (*SD* = 0.08) and with low CO values 0.20 (*SD* = 0.09). In scenario 2 the mean for team performance was 6.26 (*SD* = 2.51) for teams with high CO values and it was 4.36 (*SD* = 2.24) for teams with low CO values. The mean concerning time without water for teams with high CO values was 0.18 (*SD* = 0.08) and with low CO values 0.25 (*SD* = 0.11).

For analyzing indirect effects, CO was the independent variable, time without water the mediator and team performance the dependent variable. The findings indicated that CO has an indirect effect on team performance mediated by time without water for scenario 1 (Table 4) and scenario 2 (Table 5). In scenario 1, CO had no direct effect on team performance (*b(YX)*), but CO significantly predicted time without water (*b(MX)*). A significant total effect (*b(YX)*) is not an assumption in the assessment of indirect effects, and therefore the non-significance of this relationship does not violate the analysis (see Preacher and Hayes, 2004, p. 719). Furthermore, time without water significantly predicted team performance when controlling for CO (*b(YM.X)*), whereas the effect of CO on team performance was not significant when controlling for time without water (*b(YX.M)*). The indirect effect was 0.40 and significant when using normal distribution and estimated with the Sobel test (*z* = 1.97, *p* < 0.05). The bootstrap procedure was applied to estimate the effect size not based on the assumption of normal distribution. As displayed in Table 4, the bootstrapped estimate of the indirect effect was 0.41 and the true indirect effect was estimated to lie between 0.0084 and 0.9215 with a 95% confidence interval. As zero is not in the 95% confidence interval, it can be concluded that the indirect effect is indeed significantly different from zero at *p* < 0.05 (two-tailed).

Table 4Indirect Effect for Coordination and Team Performance in Scenario 1.

**Table:** 

**Effects**	**Coefficient**	**SE**	***T*-ratio**
b (YX)	00.5921	0.4047	1.4630
b (MX)	−00.0365	0.0171	−2.1329^*^
b (YM.X)	−10.9712	1.9735	−5.5592^**^
b (YX.M)	00.1920	0.3673	0.5228

**Table:** 

**Indirect Effect and Significance Using Normal Distribution**						
	**Value**	**SE**	**LL 95 CI**	**UL 95 CI**	***Z***
Sobel	0.4000	0.2037	0.0008	0.7993	1.9693^*^

**Table:** 

**Bootstrap Results for Indirect Effect**						
	**Mean**	**SE**	**LL 95 CI**	**UL 95 CI**	**LL 99 CI**	**UL 99 CI**
Effect	0.4134	0.2346	0.0084	0.9215	−0.0924	1.0999

*Y = Team Performance Scenario 1; X = Collective Orientation T0; M = Coordination (time without water in scenario 1); Number of Bootstrap Resamples 5000*.

* *p < 0.05*,

** *p < 0.01*.

Table 5Indirect Effect for Coordination and Team Performance in Scenario 2.

**Table:** 

**Effects**	**Coefficient**	**SE**	***T*-ratio**
b (YX)	1.1086	0.4999	2.2176^*^
b (MX)	−0.0915	0.0185	−4.9419^**^
b (YM.X)	−6.5735	2.4634	−2.6685^**^
b (YX.M)	0.5071	0.5366	0.9450

**Table:** 

**Indirect Effect and Significance Using Normal Distribution**						
	**Value**	**SE**	**LL 95 CI**	**UL 95 CI**	***Z***
Sobel	0.6015	0.2602	0.0915	1.1115	2.3117^*^

**Table:** 

**Bootstrap Results for Indirect Effect**						
	**Mean**	**SE**	**LL 95 CI**	**UL 95 CI**	**LL 99 CI**	**UL 99 CI**
Effect	0.6055	0.2324	0.1876	1.1014	0.0340	1.2578

*Y = Team Performance Scenario 2; X = Collective Orientation T0; M = Coordination (time without water in scenario 2); Number of Bootstrap Resamples 5000*.

* *p < 0.05*,

** *p < 0.01*.

Regarding scenario 2, CO had a direct effect on team performance (*b(YX)*) and on time without water (*b(MX)*). Again, time without water significantly predicted team performance when controlling for CO (*b(YM.X)*), whereas the effect of CO on team performance was not significant when controlling for time without water (*b(YX.M)*). This time, the indirect effect was 0.60 (Sobel test, *z* = 2.31, *p* < 0.05). As displayed in Table 5, the bootstrapped estimate of the indirect effect was 0.61 and the true indirect effect was estimated to lie between 0.1876 and 1.1014 with a 95% confidence interval and between 0.0340 and 1.2578 with a 99% confidence interval. Because zero is not in the 99% confidence interval, it can be concluded that the indirect effect is indeed significantly different from zero at *p* < 0.01 (two-tailed).

**Figure 6:**
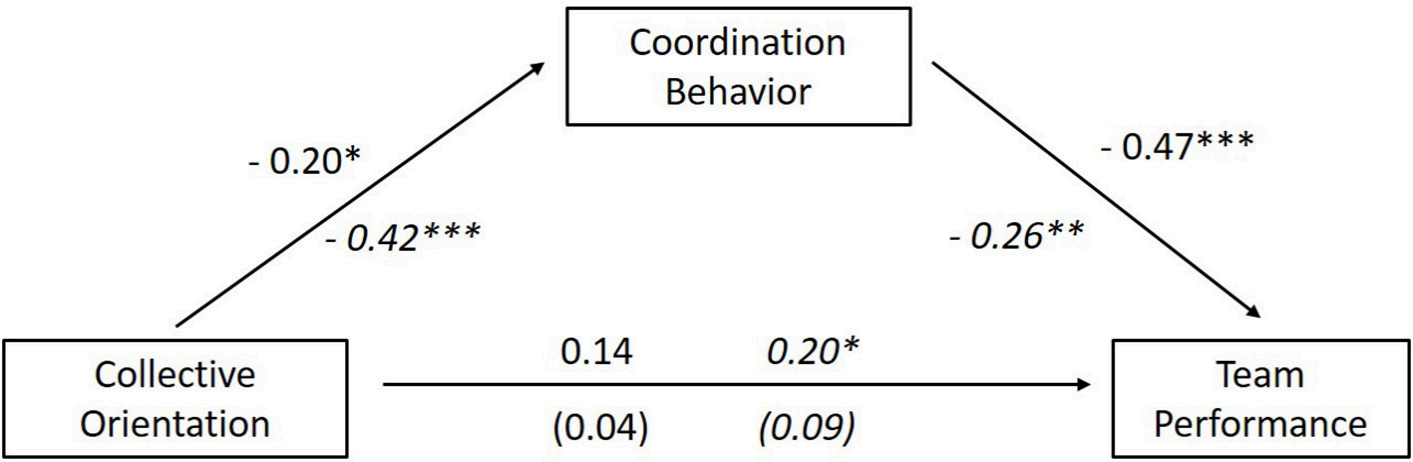
Indirect effect of collective orientation on team performance via coordination within the teams for scenario 1 and 2, ^*^p < 0.05, ^**^p < 0.01, ^***^p < 0.001, numbers in italic represent results from scenario 2, non-italic numbers are from scenario 1.

The indirect effects for both scenarios are visualized in Figure 6. Summing up, the results support hypothesis 1 and indicate that CO has an indirect effect on team performance mediated by the teams' coordination behavior, an action process. That means, fulfilling team process demands affect the dynamic decision making quality of teams acting in complex situations and input variables such as CO influence the action processes within teams positively.

Trust between team members assessed after scenario 1 (T1) and after scenario 2 (T2) did not show any significant correlation with the coordination behavior or with team complex problem solving in scenarios 1 and 2 (Table 3). Thus, hypotheses 2a and 2b are not supported. Cohesion at T1 showed no significant relationship with team performance in both scenarios, one significant negative correlation (*r* = −0.22, *p* < 0.05) with the coordination behavior in scenario 1 and no correlation with the coordination behavior in scenario 2. Cohesion at T2 did not show any significant correlation with the coordination behavior or with team performance in both scenarios. Thus, hypotheses 3a and 3b could also not be supported. Furthermore, the results showed no significant relations between CO and trust and cohesion. The correlations between trust and cohesion ranged between *r* = 0.39 and *r* = 0.51 (*p* < 0.01).

## Discussion

The purpose of our paper was first to give a sound theoretical overview and to combine theoretical approaches about team competencies and team process demands in collaborative complex problem solving and second to demonstrate the importance of selected team competencies and processes on team performance in complex problem solving by means of results from a laboratory study. We introduced the model of an idealized teamwork process that complex problem solving team pass through and integrated the relevant teamwork skills for interdependently working teams into it. Moreover, we highlighted the episodic aspect concerning complex problem solving in teams and combined the well-known transition, action, interpersonal and learning processes of teamwork with the idealized teamwork process model. Finally, we investigated the influence of trust, cohesion, and CO on action processes, such as coordination behavior of complex problem solving teams and on team performance.

Regarding hypothesis 1, studies have indicated that teams whose members have high CO values are more successful in their coordination processes and task accomplishment (Eby and Dobbins, 1997; Driskell et al., 2010; Hagemann, 2017), which may enhance team performance through considering task inputs from other team members, information sharing and strategizing (Salas et al., 2005). Thus, we had a close look on CO as an emergent state in the present study, because emergent states support the execution of behavioral processes. In order to analyze this indirect effect of CO on team performance via coordination processes, we used the time, which firefighters spent without water in a scenario, as an indicator for high-quality coordination within the team. A small amount of time without water represents sharing information and resources between team members in a reciprocal manner, which are essential qualities of effective coordination (Ellington and Dierdorff, 2014). One of the two team members was in charge of the mobile water tank unit and therefore responsible for filling up the water tanks of his/her own firefighting unit and that of the other team member on time. In order to avoid running out of water for firefighting, the team members had to exchange information about, for example, their firefighting units' current and future positions in the field, their water levels, their strategies for extinguishing one or two fires, and the water tank unit's current and future position in the field. The simple mediation models showed that CO has an indirect effect on team performance mediated by time without water, supporting hypothesis 1. Thus, CO facilitates high-quality coordination within complex problem solving teams and this in turn influences decision-making and team performance positively (cf. Figure 1). These results support previous findings concerning the relationships between emergent states, such as CO, and the team process, such as action processes like coordination (Cannon-Bowers et al., 1995; Driskell et al., 2010) and between the team process and the team performance (Stevens and Campion, 1994; Dierdorff et al., 2011).

Hypotheses 2 and 3 analyzed the relationships between trust and cohesion and coordination and team performance. Because no correlations between trust and cohesion and the coordination behavior and team complex problem solving existed, further analyses, like mediation analyses, were unnecessary. In contrast to other studies (McAllister, 1995; Beal et al., 2003; Salas et al., 2005; Kozlowski and Ilgen, 2006), the present study was not able to detect effects of trust and cohesion on team processes, like action processes, or on team performance. This can be attributed to the restricted sample composition or the rather small sample size. Nevertheless, effect sizes were small to medium, so that they would have become significant with an increased sample sizes. The prerequisite, mentioned by the authors, that interdependence of the teamwork is important for identifying those effects, was given in the present study. Therefore, this aspect could not have been the reason for finding no effects concerning trust and cohesion. Trust and cohesion within the teams developed during working on the simulation scenarios while fighting fires, showed significant correlations with each other, and were unrelated to CO, which showed an effect on the coordination behavior and the team performance indeed. The results seem to implicate, that the influence of CO on action processes and team performance might be much more stronger than those of trust and cohesion. If these results can be replicated should be analyzed in future studies.

As the interdependent complex problem-solving task was a computer-based simulation, the results might have been affected by the participants' attitudes to using a computer. For example, computer affinity seems to be able to minimize potential fear of working with a simulation environment and might therefore, be able to contribute to successful performance in a computer-based team task. Although computers and other electronic devices are pervasive in present-day life, computer aversion has to be considered in future studies within complex problem-solving research when applying computer-based simulation team tasks. As all of the participants were studying applied cognitive science, which is a mix of psychology and computer science, this problem might not have been influenced the present results. However, the specific composition of the sample reduces the external validity of the study and the generalizability of the results. A further limitation is the small sample size, so that moderate to small effects are difficult to detect.

Furthermore, laboratory research of teamwork might have certain limitations. Teamwork as demonstrated in this study fails to account for the fact that teams are not simple, static and isolated entities (McGrath et al., 2000). The validity of the results could be reduced insofar as the complex relationships in teams were not represented, the teamwork context was not considered, not all teammates and teams were comparable, and the characteristic as a dynamic system with a team history and future was not given in the present study. This could be a possible explanation why no effects of trust and cohesion were found in the present study. Maybe, the teams need more time working together on the simulation scenarios in order to show that trust and cohesion influence the coordination with the team and the team performance. Furthermore, Bell (2007) demonstrated in her meta-analysis that the relationship between team members' attitudes and the team's performance was proven more strongly in the field compared to the laboratory. In consideration of this fact, the findings of the present study concerning CO are remarkable and the simulation based microworld C3Fire (Granlund et al., 2001; Granlund, 2003) seems to be appropriate for analyzing complex problem solving in interdependently working teams.

An asset of the present study is, that the teams' action processes, the coordination performance, was assessed objectively based on logged data and was not a subjective measure, as is often the case in group and team research studies (cf. Van de Ven et al., 1976; Antoni and Hertel, 2009; Dierdorff et al., 2011; Ellington and Dierdorff, 2014). As coordination was the mediator in the analysis, this objective measurement supports the validity of the results.

### Outlook

As no transition processes such as mission analysis, formulation, and planning (Prince and Salas, 1993), goal specification (Prussia and Kinicki, 1996), and strategy formulation (Prince and Salas, 1993; Cannon-Bowers et al., 1995) as well as action processes such as monitoring progress toward goals (Cannon-Bowers et al., 1995) and systems monitoring (Fleishman and Zaccaro, 1992) were analyzed within the present study, future studies should collect data concerning these processes in order to show their importance on performance within complex problem solving teams. Because these processes are difficult to observe, subjective measurements are needed, for example asking the participants after each scenario how they have prioritized various tasks, if and when they have changed their strategy concerning protecting houses or fighting fires, and on which data within the scenarios they focused for collecting information for goal and systems monitoring. Another possibility could be using eye-tracking methods in order to collect data about collecting information for monitoring progress toward goals, e.g., collecting information how many cells are still burning, and systems monitoring, e.g., tracking team resources like water for firefighting.

CO is an emergent state and emergent states can be influenced by experience or learning, for example (Kozlowski and Ilgen, 2006). Learning processes (Edmondson, 1999), that Schmutz et al. (2016) added to the taxonomy of team processes developed by Marks et al. (2001) and which occur during transition and action phases and contribute to team effectiveness include e.g., *feedback*. Feedback can be useful for team learning when team learning is seen as a form of information processing (Hinsz et al., 1997). Because CO supports action processes, such as coordination and it can be influenced by learning, learning opportunities, such as feedback, seem to be important for successful task accomplishment and for supporting teams in handling complex situations or problems. If the team is temporarily and interpersonally unstable, as it is the case for most of the disaster or crisis management teams dealing with complex problems, there might be less opportunities for generating shared mental models by experiencing repetitive cycles of joint action (cf. Figure 2) and strategies such as cross training (Salas et al., 2007) or feedback might become more and more important for successful complex problem solving in teams. Thus, for future research it would be of interest to analyze what kind of feedback is able to influence CO positively and therefore is able to enhance coordination and performance within complex problem-solving teams.

Depending on the type of feedback, different main points will be focused during the feedback (see Gabelica et al., 2012). Feedback can be differentiated into performance and process feedback. Process feedback can be further divided into task-related and interpersonal feedback. Besides these aspects, feedback can be given on a team-level or an individual-level. Combinations of the various kinds of feedback are possible and are analyzed in research concerning their influence on e.g., self- and team-regulatory processes and team performance (Prussia and Kinicki, 1996; Hinsz et al., 1997; Jung and Sosik, 2003; Gabelica et al., 2012). For future studies it would be relevant to analyze, whether it is possible to positively influence the CO of team members and therefore action processes such as coordination and team performance or not. A focus could be on the learning processes, especially on feedback, and its influence on CO in complex problem solving teams. So far, no studies exist that analyzed the relationship between feedback and a change in CO, even though researchers already discuss the possibility that team-level process feedback shifts attention processes on team actions and team learning (McLeod et al., 1992; Hinsz et al., 1997). These results would be very helpful for training programs for fire service or police or medical teams working in complex environments and solving problems collaboratively, in order to support their team working and their performance.

In summary, the idealized teamwork process model is in combination with the transition, action, interpersonal and learning processes a good framework for analyzing the impact of teamwork competencies and teamwork processes in detail on team performance in complex environments. Overall, the framework offers further possibilities for investigating the influence of teamwork competencies on diverse processes and teamwork outcomes in complex problem solving teams than demonstrated here. The results of our study provide evidence of how CO influences complex problem solving teams and their performance. Accordingly, future researchers and practitioners would be well advised to find interventions how to influence CO and support interdependently working teams.

## Ethics statement

This study was carried out in accordance with the recommendations of Ethical guidelines of the German Association of Psychology, Ethics committee of the University of Duisburg-Essen, Department of Computer Science and Applied Cognitive Science with written informed consent from all subjects. All subjects gave written informed consent in accordance with the Declaration of Helsinki. The protocol was approved by the Ethics committee of the University of Duisburg-Essen, Department of Computer Science and Applied Cognitive Science.

## Author contributions

VH and AK were responsible for the conception of the work and the study design. VH analyzed and interpreted the collected data. VH and AK drafted the manuscript. They approved it for publication and act as guarantors for the overall content.

### Conflict of interest statement

The authors declare that the research was conducted in the absence of any commercial or financial relationships that could be construed as a potential conflict of interest.
