# Supplementary material for: Complex Problem Solving in Teams: The Impact of Collective Orientation on Team Process Demands
Source: Front Psychol. 2017 Sep 29;8:1730. doi: 10.3389/fpsyg.2017.01730 (PMC5627219; doi:10.3389/fpsyg.2017.01730)
Supplement: Supplementary file 1 [file Table1.DOCX]

Supplementary Material

Complex Problem Solving in Teams: The Impact of Collective Orientation on Team Process Demands

Vera Hagemann*, Annette Kluge

*** Correspondence:** Vera Hagemann: vera.hagemann@rub.de


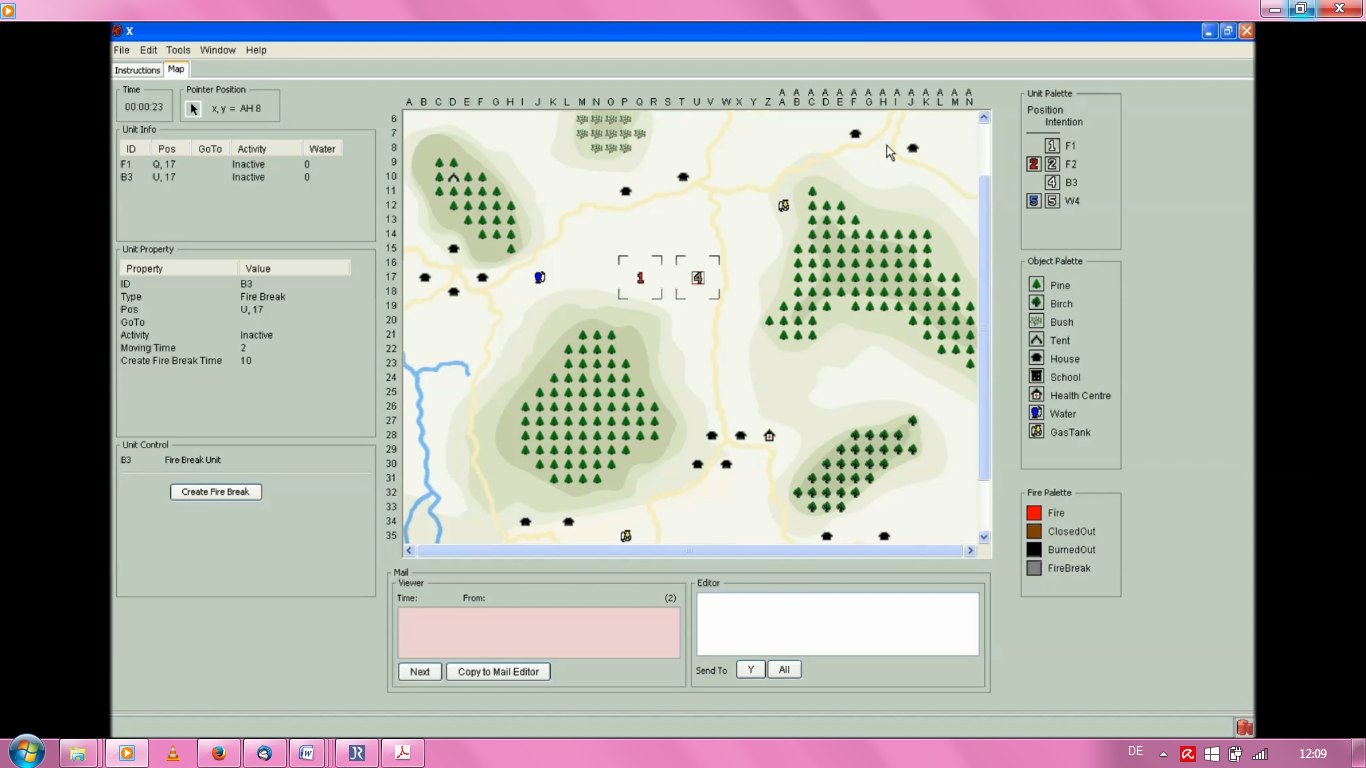


**Supplementary Figure 1.** User interface of C³Fire simulation (participants’ point of view).


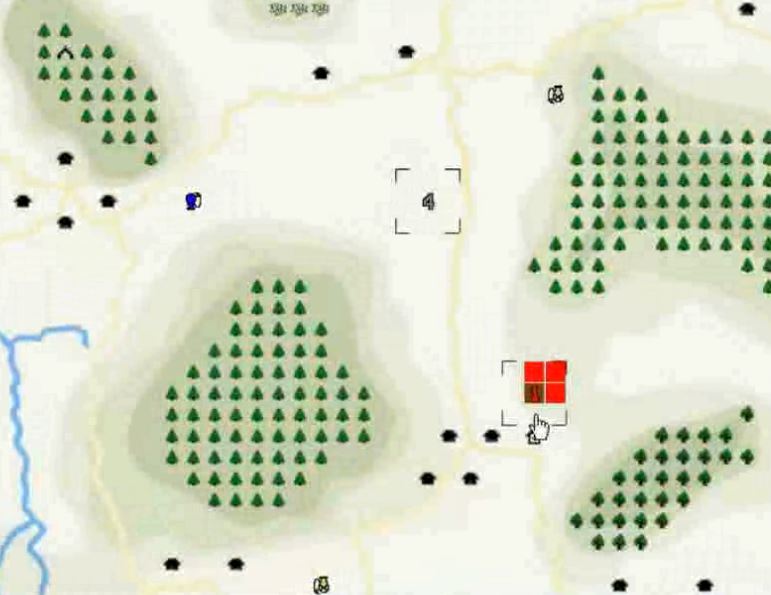


**Supplementary Figure 2.** Detail of the user interface for one team member (grey 4=fire-break unit, red 1=fire-fighting unit, 15 black houses, two yellow gas tanks, one blue local water-tank, one black tent (top left), one hospital (under curser), red field=fire, brown field=extinguished).


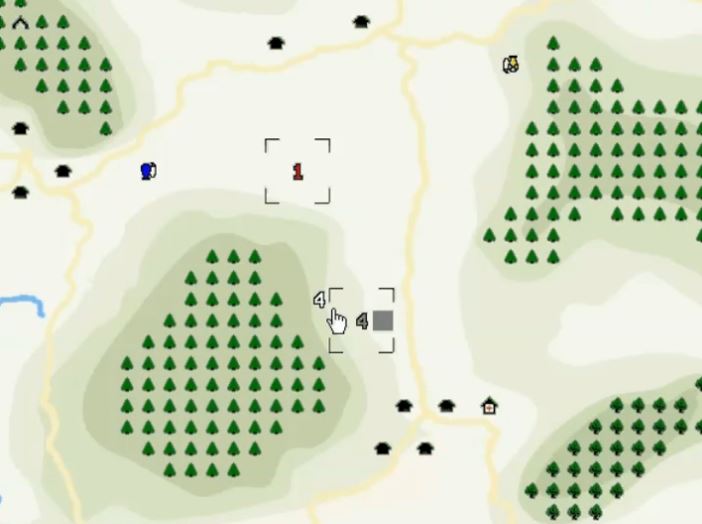


**Supplementary Figure 3.** Actions of the fire-break unit (grey 4=fire-break unit current position, white 4=fire-break unit future position, grey field=placed fire-break).

| **Characteristic** | **Scenario 1** | **Scenario 2** |
| --- | --- | --- |
| Size of visibility field | 3 x 3 | 3 x 3 |
| Number of fires | 2 | 2 |
| Number of cells for fire break-out 1 | 1 | 1 |
| Number of cells for fire break-out 2 | 2 | 2 |
| Time fire break-out 1 (after min:sec) | 01:30 | 01:30 |
| Time fire break-out 2 (after min:sec) | 06:30 | 06:30 |
| Place of fire break-out 1 | Normal grass | Normal grass |
| Place of fire break-out 2 | Normal grass (both cells) | Normal grass (both cells) |
| Water filling level for fire-fighting units at the beginning in liters | 0 | 0 |
| Mobile water-tank unit’s filling level at the beginning in liters | 180 | 180 |
| Distance between local water-tank and fire-fighting units and mobile water-tank unit | close | afar |
| First advice (after min:sec): All units! Be prepared for the mission. | 00:00 | 00:00 |
| Second advice (after min:sec): Take care that your water tanks are filled. | 00:15 | 00:15 |
| Third advice only for one player (after min:sec): Take care that all fire-fighting units are supported with water for the whole time. | 00:30 | 00:30 |
| Advice for location fire 1 (after min:sec) | 02:30 | 02:30 |
| Advice for location fire 2 (after min:sec) | 06:45 | 06:45 |
| Wind direction | East | West |
| Wind speed | 5.0 (medium speed) | 5.0 (medium speed) |
| Burning rate normal | 1,5 | 1,5 |
| Burning rate for pines | 1 | 1 |
| Burning rate for birches | 1 | 1 |
| Burning rate for bushes | 0,5 | 0,5 |
| Running speed (sec for one cell) | 2 | 2 |
| Extinction time for one cell in sec | 5 | 5 |
| Ignition time for one cell in sec | 60 sec | 60 sec |
| Burning duration for one cell until burnt out in sec | 120 sec | 120 sec |
| Mobile water-tank unit contains water for | 3 units (180) | 3 units (180) |
| Amount of water for one fire-fighting unit in liters | 60 | 60 |
| Water use to extinguish one cell in liters | 5 | 5 |
| Start position: Fire-fighting unit 1 | 19 , 21 | 19 , 21 |
| Start position: Fire-fighting unit 2 | 21 , 21 | 21 , 21 |
| Start position: Fire-break unit | 19 , 23 | 19 , 23 |
| Start position: Water-tank unit | 12 , 23 | 12 , 23 |
| Delay both fire-fighting units at the same cell | 12 sec | 12 sec |
| Number of houses | 15 | 15 |
| Number of tents | 1 | 1 |
| Number of schools | 1 | 1 |
| Number of hospitals | 1 | 1 |
| Number of gas tanks | 2 | 2 |
| Cells burning | red | red |
| Cells burnt down | black | black |
| Cells extinguished | brown | brown |
| Communication | e-mail / Chat | e-mail / Chat |
| Peculiarities of scenarios |  | Water tank is far away |

**Supplementary Table 1.** Detailed information regarding the C³Fire scenarios’ characteristics and differences.
